# A TRPC3/6 Channel Inhibitor Promotes Arteriogenesis after Hind-Limb Ischemia

**DOI:** 10.3390/cells11132041

**Published:** 2022-06-27

**Authors:** Tsukasa Shimauchi, Takuro Numaga-Tomita, Yuri Kato, Hiroyuki Morimoto, Kosuke Sakata, Ryosuke Matsukane, Akiyuki Nishimura, Kazuhiro Nishiyama, Atsushi Shibuta, Yutoku Horiuchi, Hitoshi Kurose, Sang Geon Kim, Yasuteru Urano, Takashi Ohshima, Motohiro Nishida

**Affiliations:** 1 National Institute for Physiological Sciences (NIPS), National Institutes of Natural Sciences, Okazaki 444-8585, Japan; tsukasa.shimauchi.1@ulaval.ca (T.S.); ta96tomita@shinshu-u.ac.jp (T.N.-T.); aki@nips.ac.jp (A.N.); 2 Exploratory Research Center on Life and Living Systems (ExCELLS), National Institutes of Natural Sciences, Okazaki 444-8787, Japan; 3 Graduate School of Pharmaceutical Sciences, Kyushu University, Fukuoka 812-8582, Japan; yu-kato@phar.kyushu-u.ac.jp (Y.K.); hmorimoto@phar.kyushu-u.ac.jp (H.M.); sakata.kosuke.243@s.kyushu-u.ac.jp (K.S.); matsukane.ryosuke.096@m.kyushu-u.ac.jp (R.M.); knishiyama@phar.kyushu-u.ac.jp (K.N.); s.b.ch0910@gmail.com (A.S.); yutoku328@gmail.com (Y.H.); hitoshikurose1436@gmail.com (H.K.); ohshima@phar.kyushu-u.ac.jp (T.O.); 4 Department of Molecular Pharmacology, Shinshu University School of Medicine and Health Sciences, Matsumoto 390-8621, Japan; 5 Department of Physiological Sciences, SOKENDAI (School of Life Science, The Graduate University for Advanced Studies), Okazaki 444-8585, Japan; 6 College of Pharmacy, Dongguk University-Seoul, Goyang-si 10326, Gyeonggi-Do, Korea; sgk@snu.ac.kr; 7 Laboratory of Chemistry and Biology, Graduate School of Pharmaceutical Sciences, The University of Tokyo, Tokyo 113-0033, Japan; uranokun@m.u-tokyo.ac.jp

**Keywords:** canonical transient receptor potential 6, peripheral arterial disease, vessel maturation, 1-benzilpiperadine

## Abstract

Retarded revascularization after progressive occlusion of large conductance arteries is a major cause of bad prognosis for peripheral artery disease (PAD). However, pharmacological treatment for PAD is still limited. We previously reported that suppression of transient receptor potential canonical (TRPC) 6 channel activity in vascular smooth muscle cells (VSMCs) facilitates VSMC differentiation without affecting proliferation and migration. In this study, we found that 1-benzilpiperadine derivative (1-BP), a selective inhibitor for TRPC3 and TRPC6 channel activities, induced VSMC differentiation. 1-BP-treated mice showed increased capillary arterialization and improvement of peripheral circulation and skeletal muscle mass after hind-limb ischemia (HLI) in mice. 1-BP had no additive effect on the facilitation of blood flow recovery after HLI in TRPC6-deficient mice, suggesting that suppression of TRPC6 underlies facilitation of the blood flow recovery by 1-BP. 1-BP also improved vascular nitric oxide bioavailability and blood flow recovery after HLI in hypercholesterolemic mice with endothelial dysfunction, suggesting the retrograde interaction from VSMCs to endothelium. These results suggest that 1-BP becomes a potential seed for PAD treatments that target vascular TRPC6 channels.

## 1. Introduction

Peripheral arterial disease (PAD) represents impairment of lower extremity blood flow caused by arteriosclerosis progression. PAD patients are complicated with intermittent claudication, and decreased physical activity with inadequate blood flow to meet the metabolic demand of the muscle [1]. Impairment of physical activity is associated with cardiovascular diseases, such as coronary artery disease and cerebral artery disease [2,3]. PAD is also the main cause of amputation, and recognized as a personal, social, and economic burden worldwide [4,5,6]. However, according to clinical evidence, approved medical therapies for PAD are not efficacious enough to prevent poor prognosis [7].

The transient receptor potential (TRP) channel was identified in a Drosophila visual transduction mutation [8]. Because of their many functions in sensory neuron, immunity, respiration, and circulation, TRP channels are paid attention as therapeutic targets [9]. The canonical TRP subfamily (TRPC) proteins form voltage-independent, non-selective cation channel activated downstream of phospholipase C, and are composed of seven subtypes of TRPC1 to C7. Among them, TRPC3 and TRPC6 have a critical role in the regulation of cardio-circulatory signaling, such as cardiac hypertrophy induced by angiotensin II [10], permeability of vascular endothelial cells [11], and pulmonary hypertension [12,13]. We previously reported that cilostazol, the most effective clinical drug for PAD patients, suppresses the TRPC6 channel via phosphorylation of threonine 69 which leads to vasodilation [14]. Furthermore, the genetic ablation of *trpc6* facilitates vascular smooth muscle cell (VSMC) differentiation without affecting cell proliferation and migration properties in primary-cultured mouse aortic VSMCs, which is necessary for the facilitation of arteriogenesis after hindlimb ischemia (HLI) [15,16]. These facts now prompted us to investigate the possibility of TRPC3/6 inhibition as a novel therapeutic target for PAD.

In this study, we identified a TRPC3/6 inhibitor significantly improved blood flow recovery during HLI, whereas TRPC6 knockout mice were completely insensitive to the agent. Strikingly, the molecule exhibited blood flow improvement even in endothelial dysfunction model mice. Our findings strongly implicate that direct inhibition of the TRPC3/6 channel can be a novel therapeutic strategy for PAD independently of endothelial function.

## 2. Materials and Methods

### 2.1. Synthesis of 1-BP (1-Benzyl-1-(11-hydroxyundecyl)piperidin-1-ium chloride)

1-BP was synthesized as shown below. To the test tube equipped with a stir bar, 11-(piperidin-1-yl)undecan-1-ol (127.7 mg, 0.50 mmol; synthesized from 11-bromoundecan-1-ol (Kanto Chemical, Tokyo, Japan) and piperidine (Kishida Chemical, Osaka, Japan) according to the procedure described in EP493970 A1, 1992) and toluene (1.5 mL, FUJIFILM Wako, Osaka, Japan) were added before the addition of benzyl chloride (115 µL, 1.0 mmol, 2.0 equiv, Kishida Chemical, Osaka, Japan), and the mixture was refluxed with stirring for 18 h. After cooling to room temperature, the volatiles were removed in vacuo, and the crude solid was filtered, washed with ethyl acetate (FUJIFILM Wako, Osaka, Japan), and dried under vacuum to give 1-BP (as the chloride salt) as a white solid (83.6 mg, 44% yield).

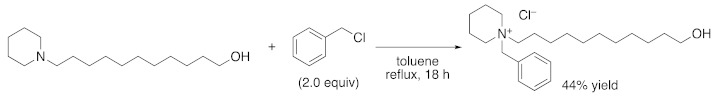



1-BP (1-Benzyl-1-(11-hydroxyundecyl)piperidin-1-ium chloride) mp. 150.7–151.2 °C. ^1^H NMR (500 MHz, CDCl_3_) δ 7.66–7.54 (m, 2H), 7.52–7.38 (m, 3H), 5.00 (s, 2H), 4.00 (brdd, *J* = 11.6, 10.9 Hz, 2H), 3.64 (t, *J* = 6.6 Hz, 2H), 3.51 (brd, *J* = 12.7 Hz, 2H), 3.41–3.25 (m, 2H), 2.03–1.90 (m, 2H), 1.86 (br, 1H), 1.84–1.72 (m, 6H), 1.57 (tt, *J* = 6.7, 7.0 Hz, 2H), 1.47–1.11 (m, 14H). ^13^C{^1^H} NMR (125 MHz, CDCl_3_) δ 133.1, 130.6, 129.2, 127.0, 64.4, 62.8, 57.4, 54.6, 32.7, 29.3, 29.21, 29.21, 29.15, 29.1, 26.3, 25.6, 21.9, 20.6, 20.0. FTIR (neat) 2922, 2851, 1456, 1059, 1034, 762, 712 cm^–1^. HRMS (DART) *m/z* calcd. for C_23_H_40_NO^+^ [M + H]^+^ 346.31044, found 346.31008. Other commercial TRPC channel inhibitors were purchased.

### 2.2. Cell Culture and Transfection

HEK293 cells and HeLa cells were cultured in Dulbecco’s modified eagle medium supplemented with 10% fetal bovine serum and antibiotics. Cells were transfected with plasmid DNAs encoding TRPC3, TRPC6, or TRPC7 by using X-tremeGENE™ 9 reagent (Roche, Basel, Switzerland) according to manufacturer’s instruction. Mouse aortic smooth muscle cells were isolated from aorta of 6–8 weeks old 129/Sv mice. Thoracic aorta was removed and cleaned from perivascular fat. The aorta was incubated with enzyme solution (1 mg/mL collagenase type 2 (Worthington, Columbus, OH, USA), 1 mg/mL soybean trypsin inhibitor (Sigma, St. Louis, MO, USA), and 0.75 units/mL elastase from porcine pancreas (Sigma, St. Louis, MO, USA) dissolved in Hank’s balanced salt solution (Thermo Fisher Scientific, Waltham, MA, USA) for 10 min at 37 °C. Adventitia and endothelium were then removed. Remaining medial layer was cut into small pieces and incubated with enzyme solution for 1–1.5 h at 37 °C. After trituration, cells were plated and cultured in DMEM supplemented with 20% FBS and penicillin/streptomycin at 37 °C with 5% CO_2_. Cells were used between 3rd to 7th passages. 

### 2.3. Measurement of Ca^2+^ Responses in 96-Well Plates

We used Functional Screening System 7000EX (FDSS 7000EX, Hamamatsu Photonics, Hamamatsu, Japan) to measure Ca^2+^ responses in 96-well plates for first drug screening. HeLa cells stably expressing TRPC3 were cultured for 24 h on 96-well plates (1.0 × 10^4^ cells/well). Cells were loaded with Fluo4-AM (2 μM) containing 0.2% Pluronic F-127 (Molecular Probes, Eugene, OR, USA) in Hepes-buffered salt solution (HBSS; in mM): 137 NaCl, 5.6 KCl, 2 CaCl_2_, 1 MgCl_2_, 10 Hepes, 10 glucose (pH 7.4 adjusted with NaOH) for 45 min at room temperature and then washed with HBSS. After treatment with each drug (3 μM) for 5 min, cells were stimulated with ATP (100 μM). Difference of the area under the curve (AUC) after ATP stimulation between TRPC3-expressing cells and non-TRPC3 cells were calculated as 100% and treated/control ratio less than 50% was considered significant.

### 2.4. Fluorescent Ca^2+^ Imaging

Measurement of intracellular Ca^2+^ mobilization was carried out as we previously demonstrated [14]. Briefly, the cDNA-transfected HEK293 or HeLa cells on coverslips were loaded with Fura-2 AM (1 μM, Dojindo, Kumamoto, Japan) in HBSS. Images of the Fura-2 fluorescence of the cells were recorded and analyzed with a video image analysis system (Aqua Cosmos; Hamamatsu Photonics, Hamamatsu, Japan). All the reagents dissolved in water or dimethyl sulfoxide were diluted to their final concentrations in the HBSS and applied to the cells by perfusion. The Ca^2+^-free solution contained 0.5 mM EGTA but no added CaCl_2_. Adenosine trisphosphate disodium salt, carbachol, and thapsigargin were purchased from Sigma.

### 2.5. Immunofluorescent Staining

Smooth muscle cells isolated from mouse aorta were seeded on 12 mm round coverslips and serum starved on the next day. Forty-eight hours after serum starvation, cells were fixed with 4% paraformaldehyde in phosphate-buffered saline (PBS), permeabilized with 0.5% Triton X-100 in PBS, and then incubated with anti-smooth muscle 22 α (SM22α) (Abcam, Cambridge, UK) antibody and secondary Alexa488-conjugated anti-rabbit IgG (Thermo Fisher Scientific). Coverslips were mounted on a slide glass with Prolong Diamond Antifade Mountant with 4′,6-diamidino-2-phenylindole (Thermo Fisher Scientific). Fluorescence images were acquired by fluorescence microscope with ×40 objective (BX-Z710; Keyence, Osaka, Japan). Mean fluorescence intensity/pixel of each cell (at least 30 cells/image, 3 images/reagent) was analyzed with ImageJ software (National Institute of Health, Bethesda, MD, USA).

### 2.6. Mouse Models

All protocols using BALB/c, 129/Sv background TRPC6 wild type (+/+), TRPC6 knockout (−/−), and C57BL/6 background Low density lipoprotein receptor knockout (LDLr (−/−)) mice were reviewed and approved by the ethic committees at National Institutes of Natural Sciences or the Animal Care and Use Committee, Kyushu University, and were performed according to the institutional guidelines concerning the care and handling of experimental animals. Animal studies are reported in compliance with the ARRIVE guidelines. Mice were maintained in specific-pathogen-free area at light/dark cycle of 12 h/12 h. BALB/c mice were purchased from Nihon SLC. TRPC6 (−/−) mice were provided by the Comparative Medicine Branch, National Institute of Environmental Health Sciences, Research Triangle Park, North Carolina 27709. Genotyping for TRPC6 (−/−) mice has been described previously [17]. LDLr (−/−) mice were purchased from Jackson laboratories and bred in the animal facilities. Eight- to ten-week old male mice of BALB/c and 129/Sv or LDLr (−/−) were subjected to ligation of left femoral arteries or ligation followed by excision of femoral arteries and veins, respectively. The surgery was conducted under the anesthesia with intraperitoneal injection of medetomidine (0.3 mg/kg), midazolam (4 mg/kg), and butorphanol (5 mg/kg). Mice were subcutaneously injected with 0.1 mg/kg of buprenorphine hydrochloride (Repetan; Otsuka Pharmaceutical, Chiyoda, Tokyo, Japan) as analgesia. All compounds were administered with mini-osmotic pumps model 2004 (Alzet, Cupertino, CA, USA) at the rate of 0.1 mg/kg/day 1 day before the surgery. Walking ability and feeding behavior were carefully monitored. All mice recovered walking ability a few days after the surgery and no signs of weight loss due to the defect of food and water intake. Hindlimb blood flow measurements were performed using Laser speckle imaging analyzer (Omega wave, Tokyo, Japan) under the anesthesia described above. Laser speckle image of the ischemic left and contralateral right legs were measured simultaneously and analyzed in accordance with the manufacturer’s manual. Blood flow was measured before surgery (day 0), and on days 1, 7, 14, and 21 after surgery and expressed as a percentage of ischemic left leg to non-ischemic right one. Mice were euthanized by inhalation of isoflurane overdose. 

### 2.7. Histological Analysis

The gastrocnemius muscles were harvested from mice and fixed in 4% paraformaldehyde for 24 h at 4 °C. After fixation, muscles were dehydrated in 10%, 15%, and 20% sucrose solution, embedded in optimal cutting temperature compound (Sakura finetech, Tokyo, Japan), and frozen in isopentane/dry ice. Frozen tissues were cut into 10 μm sections by cryostat Leica CM1100 (Leica, Wetzlar, Germany). After 1 h blocking with 1% bovine serum albumin, sections were stained with anti-α-smooth muscle actin (SMA) (1:500 dilution, Sigma), anti-CD31 (1:100 dilution, BioLegend, San Diego, CA, USA), anti-phospho-TRPC6 (1:100 dilution) [14] overnight at 4 °C. After washing, they were incubated with fluorescently-labeled secondary antibodies (1:500 dilution, CF488A conjugated donkey anti-mouse IgG (H+L) highly cross-adsorbed antibody for mouse, CF594 conjugated goat anti-rabbit IgG (H+L) highly cross-adsorbed antibody for rabbit, or CF594 conjugated goat anti-rat IgG (H+L) highly cross-adsorbed antibody for rat) for 1 h at room temperature. All antibodies were purchased from Biotium (Fremont, CA, USA). The specimens were observed with confocal microscope Fluoview FV10i confocal imaging system (Olympus, Tokyo, Japan) with 20× oil emersion objective lens. Analysis of the number of CD31 and α-SMA positive vessels were carried out by counting fluorescent signals in the randomly selected 20× images. Clusters of CD31 immunoreactivity were counted as an individual capillary besides the morphologically identifiable vessels with a lumen. α-SMA positive vessels were distinguished from non-specific immunoreactivity by the presence of endothelium stained by CD31 fluorescence. For the analysis of phospho-TRPC6 expression in VSMCs, fluorescence signal intensity within the region of interest (ROI) made based on α-SMA fluorescence images was quantified, which was further normalized by the signal intensity of α-SMA and represented as a fold increase from before surgery. Morphometric analysis of the gastrocnemius muscle was evaluated with hematoxylin-eosin-stained slides. The quantification of cross-sectional area (CSA) was determined at least 5 randomly chosen area (at least 100 myofiber per mouse) with ImageJ software. 

### 2.8. Analysis of Motor Activities

Physiological spontaneous activities were assessed using an animal movement analyzing system (ACTIMO-100, Shinfactory, Fukuoka, Japan). Mice were bred in a rectangular enclosure cage (30 × 20 cm) with infrared sensor at 2 cm interval and a wheel equipped to the wall to calculate the distance and the average speed of the mice. Total walking distance of the day was calculated via the addition of the floor walking and the wheel running distance. The average speed was also calculated from the running speed of floor.

### 2.9. Western Blotting

Mouse gastrocnemius muscles were homogenized in RIPA buffer containing 0.1% sodium dodecyl sulfate (SDS), 0.5% sodium deoxycholate, 1% NP-40, 150 mM NaCl, 50 mM Tris-HCl (pH 7.4) and protease inhibitor cocktail (Nacalai, Kyoto, Japan). Samples (1 mg) were fractionated by SDS-PAGE and transferred to polyvinylidene difluoride membranes (Millipore, Burlington, MA, USA). Membranes were incubated with primary antibodies of the Myoglobin (1:2000 dilution, Abcam) and β-actin (1:2000 dilution, Cell signaling, Danvers, MA, USA) overnight. After incubation of secondary antibody, proteins were detected using Western Lightning Plus ECL (PerkinElmer, Waltham, MA, USA). Images were captured with ImageQuant Las 4000 and quantified by β-actin expression levels using ImageQuant TCL software (GE healthcare Life Science, Marlborough, MA, USA).

### 2.10. Measuring Sphk-1 mRNA Expression in Gastrocnemius Muscles

RNA was isolated from frozen mouse gastrocnemius muscle samples with RNeasy Fibrous Tissue Mini Kit (Qiagen, Hilden, Germany) according to the manufacturer’s instructions. Quantitative real-time PCR was performed with ABI PRISM 7500 Real-Time PCR system (Applied Biosystems, Waltham, MA, USA) and OneStep RT-PCR kit (Qiagen, Hilden, Germany). Sphk-1 SYBR Green primers were forward 5′-GGAGGAGGCAGAGATAACCTT-3′ and reverse 5′-GACCCAACTCCTCTGCACACA-3′. Data were normalized with 18S rRNA (432930E, Applied Biosystems, Waltham, MA, USA).

### 2.11. Fluorescent Measurement of Nitric Oxide (NO)

Measurement of NO production was assessed using DCl-DA Cal AM [18]. Unfixed frozen section of gastrocnemius muscle was incubated with DCl-DA Cal AM (10 nM) for 10 min at room temperature. The specimens were exposed to PBS flow (5 mL/min for 30 min) mimicking shear stress inside vasculature. Slides were observed with confocal microscope Fluoview FV10i confocal imaging system.

### 2.12. The Tension Measurement with Arterial Rings Isolated from Mice

Mice were euthanized by the inhalation of isoflurane overdose. Immediately descending aortae were isolated then cut into approximately 1 mm width rings. The tension measurement of the aorta was performed in 37 °C Krebs solution as previously described [19]. The tension of the arteries was stimulated with phenylephrine (10 μM) followed by endothelium dependent relaxation with acetylcholine (10^−9^~10^−5^ M).

### 2.13. Electrophysiology

Carbachol-induced currents were measured using the whole-cell patch-clamp technique with an EPC-10 patch-clamp amplifier (HEKA Elektronik, Lambrecht, Germany). Patch electrodes with a resistance of 3–4 MΩ (when filled with internal solution) were made from 1.5-mm borosilicate glass capillaries (Sutter Instrument, Novato, CA, USA). Voltage-clamp experiments were performed at a holding potential of −60 mV, with recordings sampled at 2.0 kHz and filtered at 2.9 kHz. To analyze I–V relationships, ramp pulses from −100 to 100 mV over 250 milliseconds were applied every 30 s. Cells were allowed to settle in the perfusion chamber in external solution containing (in mM) 140 NaCl, 5.6 KCl, 1 MgCl_2_, 2 CaCl_2_, 10 Hepes, and 10 glucose (pH 7.4). The pipette solution contained (in mM) 120 CsOH, 120 aspartate, 20 CsCl, 2 MgCl_2_, 5 EGTA, 1.5 CaCl_2_, 10 Hepes, 2 ATP-Na_2_, 0.1 GTP, and 10 glucose (pH 7.2, adjusted with Tris base). Cells were superfused with standard external solution in the presence or absence of carbachol applied focally using a Y-tube perfusion system. Cells were treated with 1-BP (1 μM) 3 min before the carbachol application.

### 2.14. Statistics

Data analyzed by parametric tests are presented as mean ± s.e.m. and data analyzed by nonparametric analysis are presented as median with 95% confidence intervals (CI). Group size is the number of independent animals or experimental replicates. To test normal distribution of the collected data, Shapiro–Wilk normality test was applied. Comparisons of means between 2 groups were performed by unpaired Student t test and Mann-Whitney U-test for normally and not normally distributed samples, respectively. Comparisons of means among three or more groups were performed by one-way analysis of variance (ANOVA) for normally distributed samples followed by the Tukey honestly significant difference (HSD) method. Multiple comparison testing for non-normally distributed data was performed using the nonparametric Kruskal-Wallis test. For sample size less than 5-specimens per group, nonparametric statistical tests were applied. Time courses of the blood flow recovery measured by Laser speckle were analyzed by two-way ANOVA followed by the Tukey HSD method. Significance was considered at *p* < 0.05. Statistical analysis was performed using GraphPad Prism 8.0 (GraphPad Software, San Diego, CA, USA).

## 3. Results

### 3.1. Identification of Selective TRPC3/6 Inhibitors

**Figure 1:**
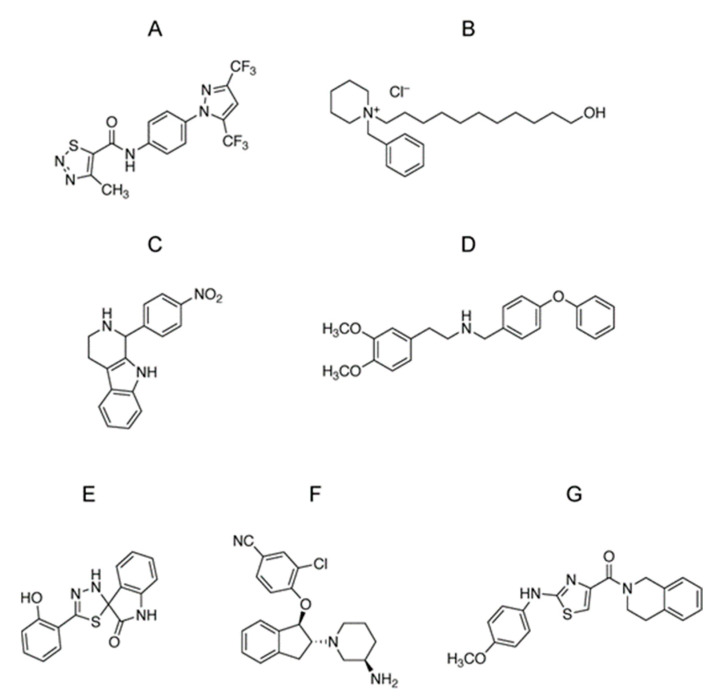
Chemical structures of TRPC3/C6 inhibitors identified in this study. Compound A, B, C, D, E, F and G are N-(4-(3,5-bis(trifluoromethyl)-1*H*-pyrazol-1-yl)phenyl)-4-methyl-1,2,3-thiadiazole-5-carboxamide (Pyr2), 1-benzyl-1-(11-hydroxyundecyl)piperidin-1-ium chloride (1-BP), 1-(4-nitrophenyl)-2,3,4,9-tetrahydro-1*H*-pyrido [3,4-*b*]indole, 2-(3,4-dimethoxyphenyl)-*N*-(4-phenoxybenzyl)ethan-1-amine, 5′-(2-hydroxyphenyl)-3′*H*-spiro[indoline-3,2′-[1,3,4]thiadiazol]-2-one, 4-(((1R,2R)-2-((R)-3-aminopiperidin-1-yl)-2,3-dihydro-1*H*-inden-1-yl)oxy)-3-chlorobenzonitrile and (3,4-dihydroisoquinolin-2(1*H*)-yl)(2-((4-methoxyphenyl)amino)thiazol-4-yl)methanone.

**Figure 2:**
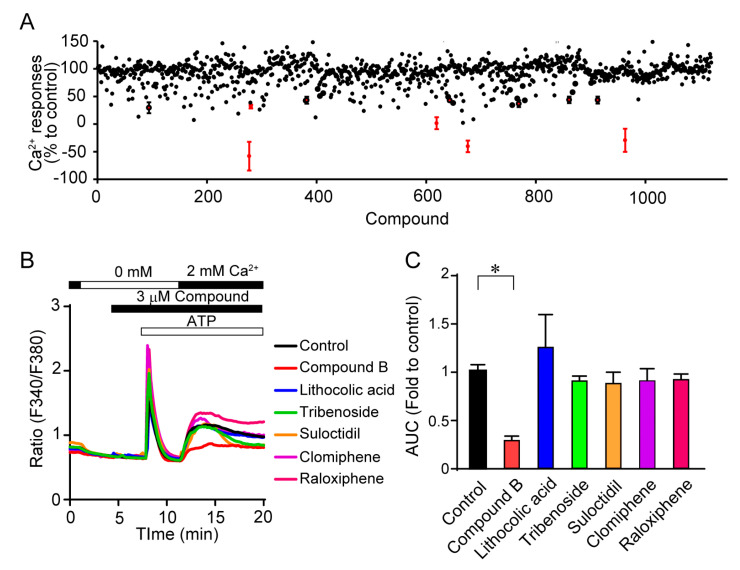
Effects of validated compounds on TRPC3-dependent increases in intracellular Ca^2+^ concentrations. (**A**) Summary of inhibitory effects of all compounds except TRPC3/6 inhibitors. The percentage of Ca^2+^ responses to control was presented. Red points with error bars were selected as hit compounds. (**B**) Representative time courses of ATP-induced Ca^2+^ responses in the absence or presence of potential TRPC3 inhibitors. (**C**) Area under the curve (AUC) of Ca^2+^ responses after Ca^2+^ add-back (n = 3 experiments). Data are mean ± s.e.m. * *p* < 0.05. Comparisons were made using One-way ANOVA followed by Tukey’s multiple comparison tests.

**Figure 3:**
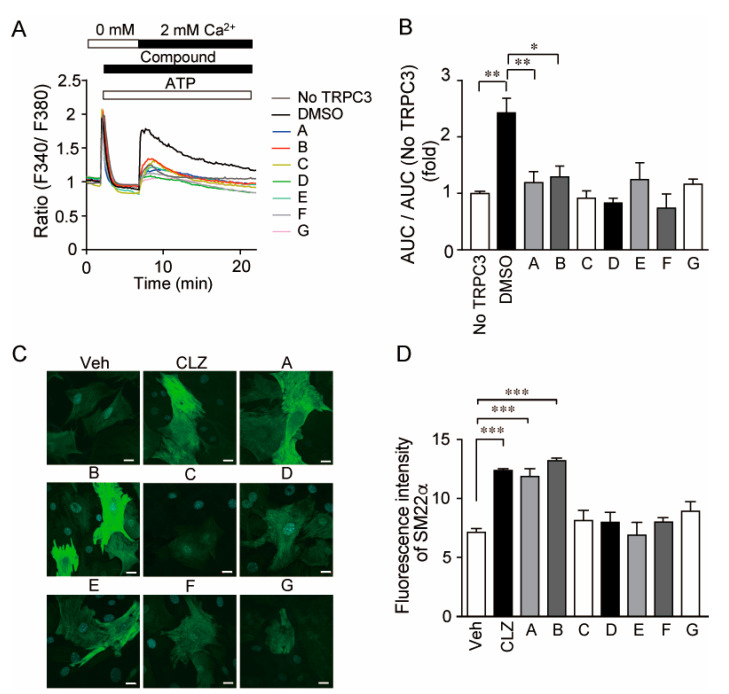
1 Effects of TRPC3/6 channel inhibitions on TRPC3-mediated Ca^2+^ influx and VSMC differentiation (**A**) Average time-dependent changes in intracellular Ca^2+^ concentration ([Ca^2+^]_i_) induced by purinergic receptor stimulation in the absence (0 mM) or presence of extracellular Ca^2+^ (2 mM) in vector (control) or TRPC3-expressing HeLa cells. Cells were stimulated with ATP (100 μM) and either control (DMSO) or each compound (1 μM). (**B**) Effects of compounds on Ca^2+^ entry-dependent [Ca^2+^]_i_ increases (n = 3 experiments). Area under the curve (AUC) was calculated by subtracting the minimal [Ca^2+^]_i_ just before the addition of external Ca^2+^ from [Ca^2+^]_i_ increases after Ca^2+^ addition every 10 s and integrating them from 7 to 22 min. (**C**) Representative fluorescent images of SM22α in mouse aortic SMCs treated with TRPC3/6 inhibitors (1 μM, compound A–G) or Cilostazol (30 μM). Compound A–G are shown in Figure 1. Cells were treated with each compound for 48 h in the absence of serum. Scale bars: 20 μm. (**D**) Quantification of fluorescence intensity of SM22α (n = 3 experiments). Data are mean ± s.e.m. * *p* < 0.05, ** *p* < 0.01, *** *p* < 0.001. Comparisons were made using One-way ANOVA followed by Tukey’s multiple comparison tests (**B**,**D**).

We first investigated the TRPC3/6 channel inhibitory actions of 1120 compounds with a HeLa cell line stably expressing TRPC3 which is closely related to TRPC6 homologue. The 1120 compounds include the validated chemical library (1088) at Open Innovation Center for Drug Discovery, The University of Tokyo, and 32 home-made existing drugs, including commercial TRPC channel inhibitors shown in Figure 1. Five compounds that suppressed TRPC3 channel activity by more than 50% were selected (Figure 2A). However, further verification by Ca^2+^ imaging showed that the suppressive effects of those compounds were less than that by compound B (Figure 2B,C). All commercial TRPC inhibitors (compounds A–G) potently suppressed TRPC3/6-mediated Ca^2+^ entry (Figure 3A,B). Compound A was Pyrazole2 (Pyr2), a broad TRPC inhibitor [20]. Compound B-E were identical with compound 6228–0473, 8009–5364, 2910–0498, and 5408–0428 for each [12]. Additionally, compound F was SAR7334 [21], and compound G was GSK-417651A [22]. All compounds have been already reported as TRPC6 inhibitors. We previously reported that genetic ablation of *trpc6* promotes VSMC differentiation without affecting proliferation and migration [16]. Primary mouse aortic SMCs were treated with the above TRPC3/6 inhibitors, and their differentiation was induced under serum starvation as previously described [16]. Differentiation status was analyzed by immunostaining of SM22α, a marker of VSMC differentiation (Figure 3C,D). The compound B termed 1-benzylpiperidine (1-BP) from its chemical structure increased the expression level of SM22α comparable to those of VSMCs treated with either Pyr2 or cilostazol (CLZ) (Figure 3C,D). We have shown that CLZ suppresses TRPC6 channel activity in a phosphorylation-dependent manner and is used as a positive control [14]. These data suggest that 1-BP might be a good candidate for the treatment of PAD.

### 3.2. 1-BP Specifically Suppresses TRPC3/6 Channels

**Figure 4:**
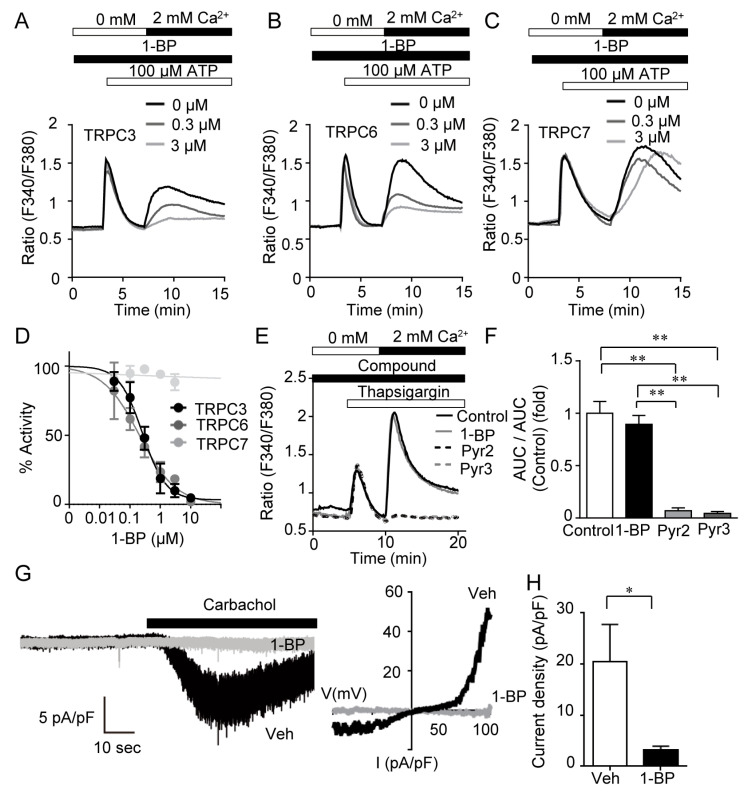
1-BP selectively suppresses TRPC3/6-mediated Ca^2+^ entry. (**A**–**C**) Average time courses of Ca^2+^ responses induced by ATP (100 μM) in HEK293 cells expressing TRPC3 (**A**), TRPC6 (**B**), and TRPC7 (**C**) (n = 3 experiments). (**D**) Concentration-dependent inhibition of 1-BP on Ca^2+^ influx-dependent [Ca^2+^]_i_ increases through TRPC channels (n = 3 experiments). (**E**) Average time courses of Ca^2+^ responses induced by thapsigargin (1 μM) in HEK293 cells. Cells were stimulated by thapsigargin (1 μM) in Ca^2+^-free external solution, and then store-operated Ca^2+^ entry (SOCE) was induced by the addition of Ca^2+^ (2 mM) (n = 3 experiments). (**F**) Inhibitory effect of DMSO (Control), 1-BP, Pyr2, and Pyr3 on SOCE-dependent [Ca^2+^]_i_ increases. AUC was calculated by subtracting the minimal [Ca^2+^]_i_ just before the addition of external Ca^2+^ from [Ca^2+^]_i_ increases after Ca^2+^ add-back every 10 s and integrating them from 10 to 20 min. (**G**) Representative continuous traces at −60 mV (left) and I–V relationships (middle) of TRPC6 current evoked by carbachol (100 μM) in HEK293 cells expressing TRPC6-EYFP. I-V relationships were obtained by subtracting currents before activation of channels from those after carbachol stimulation. (**H**) Peak current densities evoked by carbachol at the membrane potential of −60 mV in vehicle- and 1-BP-treated cells (n = 3 experiments). Data are mean ± s.e.m. * *p* < 0.05, ** *p* < 0.01.

Pharmacological characterization of 1-BP was assessed by fluorescent Ca^2+^ imaging (Figure 4A–F). At first, we examined the inhibitory effect of 1-BP on receptor-activated Ca^2+^ entry. HEK293 cells expressing TRPC channels were stimulated with ATP in the absence of extracellular Ca^2+^ followed by the addition of ATP with extracellular Ca^2+^, which presents biphasic Ca^2+^ responses. The latter Ca^2+^ responses evoked by Ca^2+^-add-back mostly represent TRPC-mediated Ca^2+^ influx. Among diacylglycerol-activated TRPC channels of -C3, -C6 and -C7 [23,24], 1-BP suppressed TRPC3 and -C6 mediated Ca^2+^ entry dose-dependently with IC_50_ of 260 nM and 206 nM, respectively (Figure 4A,B,D). In contrast, TRPC7 channel was insensitive to 1-BP (Figure 4C,D). We examined the effect of 1-BP on store-operated Ca^2+^ entry (SOCE), induced by thapsigargin in HEK293 cells. We also analyzed the effects of Pyr2 and Pyrazole3 (Pyr3), the agents known to inhibit both TRPC3/6 and SOCE. Different from Pyr2 and Pyr3, 1-BP had no suppressive effect on SOCE (Figure 4E,F). Next, we tested the effect of 1-BP on TRPC6 channel activity by electrophysiology. Carbachol, a muscarinic acetylcholine receptor agonist, evokes dually rectified non-selective cation current only in HEK293 cells expressing TRPC6 (Figure 4G). TRPC6-mediated inward currents at holding potential of −60 mV were almost completely suppressed by perfusion of 10 μM 1-BP 3 min prior to carbachol stimulation (Figure 4G,H). These results suggest that 1-BP selectively suppresses TRPC3/6 channel mediated calcium entry by inhibiting their channel activities.

### 3.3. A TRPC3/6 Inhibitor 1-BP Improves Blood Flow Recovery after HLI

**Figure 5:**
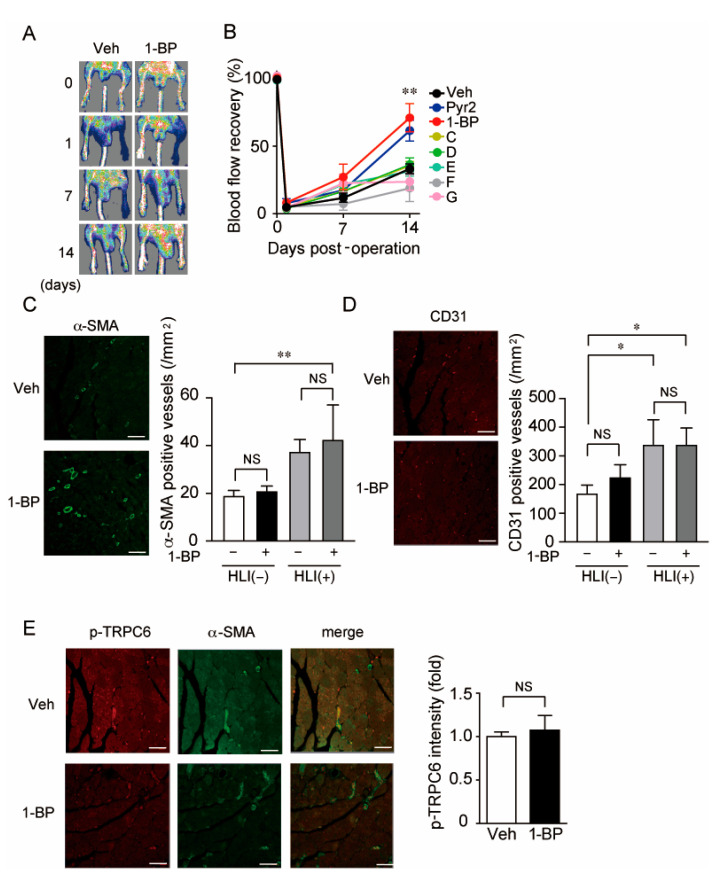
1-BP ameliorates peripheral circulation through promoting arteriogenesis after HLI. (**A**,**B**) Representative Laser Doppler images (**A**) and averaged time courses of blood flow recovery (**B**) after HLI in mice treated with seven compounds or vehicle (Veh). Osmotic pump including the respective compound (0.1 mg/kg/day) was implanted 1 day before HLI (n = 8 mice). (**C**) Representative immunohistochemical images of α-SMA in non-ischemic and ischemic gastrocnemii 2 weeks after HLI (left). The number of α-SMA-positive blood vessels in gastrocnemii (right) (n = 3 mice). Scale bars: 50 μm. (**D**) Representative immunohistochemical images of CD31 (left). The number of CD31-positive blood vessels in gastrocnemii (right) (n = 3 mice). Scale bars: 20 μm. (**E**) Representative images of Thr69-phosphorylated TRPC6 and α-SMA expressions in gastrocnemii 2 weeks after HLI (left). Quantification of phospho-TRPC6 (T69) fluorescence intensity normalized by that of α-SMA (right) (n = 3 mice). Scale bars: 50 μm. Data are mean ± s.e.m. (**B**) or median with 95% CI (C–E). * *p* < 0.05, ** *p* < 0.01, NS.; no significance. Comparisons were made using Two-way ANOVA followed by Tukey’s multiple comparison tests (**B**), One-way ANOVA followed by Tukey’s multiple comparison tests (**C**,**D**) or Mann-Whitney *U*-test (**E**).

To analyze the in vivo effect of 1-BP and other compounds on occlusive circulatory disorders, we used HLI as a mouse model of PAD by ligating the left femoral artery. Six compounds and Pyr2 were administered intraperitoneally 1 day before HLI. Only 1-BP and Pyr2, showed significant increases in blood flow recovery 2 weeks after HLI (Figure 5A,B). Gradual blood flow recovery after HLI undergoes two processes angiogenesis and arteriogenesis [25]. Angiogenesis is represented by the increase in the number of PECAM-1 (CD31) positive capillaries, and arteriogenesis is evaluated by the number of α-SMA positive vessels. Immunohistochemistry with the gastrocnemius muscle of mice 2 weeks after HLI demonstrated that the number of α-SMA positive vessels was significantly increased in the 1-BP-treated group (Figure 5C), whereas that of CD31 positive capillaries had no apparent changes in both groups (Figure 5D). These results are consistent with our previous report that the TRPC6 inhibition in SMC confers SMC differentiation and consequent peripheral circulation [16]. Furthermore, 1-BP-treated mice had no increase in TRPC6 phosphorylation levels in α-SMA positive vessels (Figure 5E), indicating that 1-BP does not suppress TRPC6 in a phosphorylation-dependent manner as does CLZ [14]. These data indicate that 1-BP suppresses the TRPC3/6 channel directly and promotes arteriogenesis rather than angiogenesis.

### 3.4. 1-BP Preserves Myoglobin Expression in Skeletal Muscle and Walking Abilities after HLI

**Figure 6:**
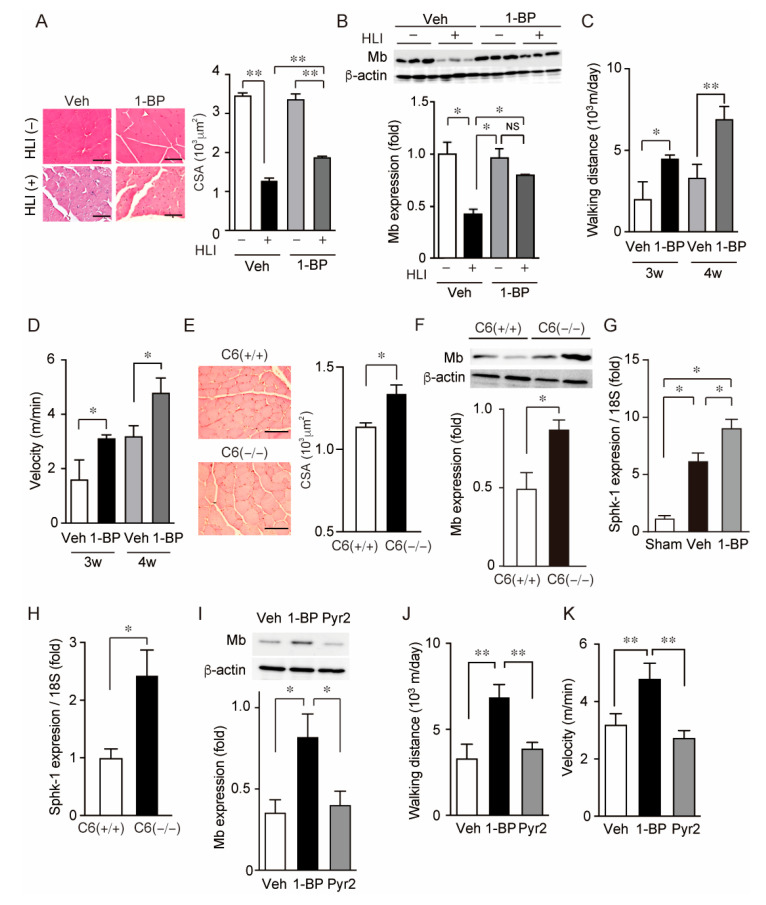
1-BP treatment preserves skeletal muscle and motor function after HLI. (**A**) Representative images of H&E staining of gastrocnemius (left). Quantification of CSA of gastrocnemius muscle fibers 3 weeks after HLI (n = 4 mice). Scale bars: 100 μm. (**B**) Representative western blots and quantification of myoglobin (Mb) abundance in gastrocnemius muscle 4 weeks after HLI (n = 3 mice). (**C**,**D**) Motor activities analyzed by averaged walking distance (**C**) and velocity (**D**) during 24 h (n = 3 mice). (**E**) Representative images of H&E staining of gastrocnemius (left). Quantification of CSA of gastrocnemius muscle fibers 3 weeks in either WT (C6 (+/+)) or TRPC6 deficient (C6(−/−)) mice after HLI (n = 4 mice). Scale bars: 100 μm. (**F**) Representative western blots and quantification of myoglobin (Mb) abundance in gastrocnemius muscle of either C6(+/+) or C6(−/−) mice 4 weeks after HLI (n = 5 mice). (**G**,**H**) Relative mRNA levels of Sphk-1 in gastrocnemius muscle 2 weeks after HLI in each compound treated mice (**G**) (n = 4 mice) and in either C6(+/+) or C6(−/−) mice (**H**) (n = 4 mice). (**I**) Representative western blots and quantification of Mb abundance in gastrocnemius muscle treated with either vehicle, 1-BP, or Pyr2 4 weeks after HLI. (n = 3 mice). (**J**,**K**) Motor activities analyzed by averaged walking distance (**J**) and velocity (**K**) for 24 h. (n = 6–11 mice). Data are median with 95% CI (**A**–**I**) or mean ± s.e.m. (**J**,**K**). * *p* < 0.05, ** *p* < 0.01. Comparisons were made using Kruskal-Wallis test (**A**,**B**,**G**,**I**), Mann-Whitney *U*-test (**C**–**F**,**H**) or One-way ANOVA followed by Tukey’s multiple comparison tests (**J**,**K**).

**Figure 7:**
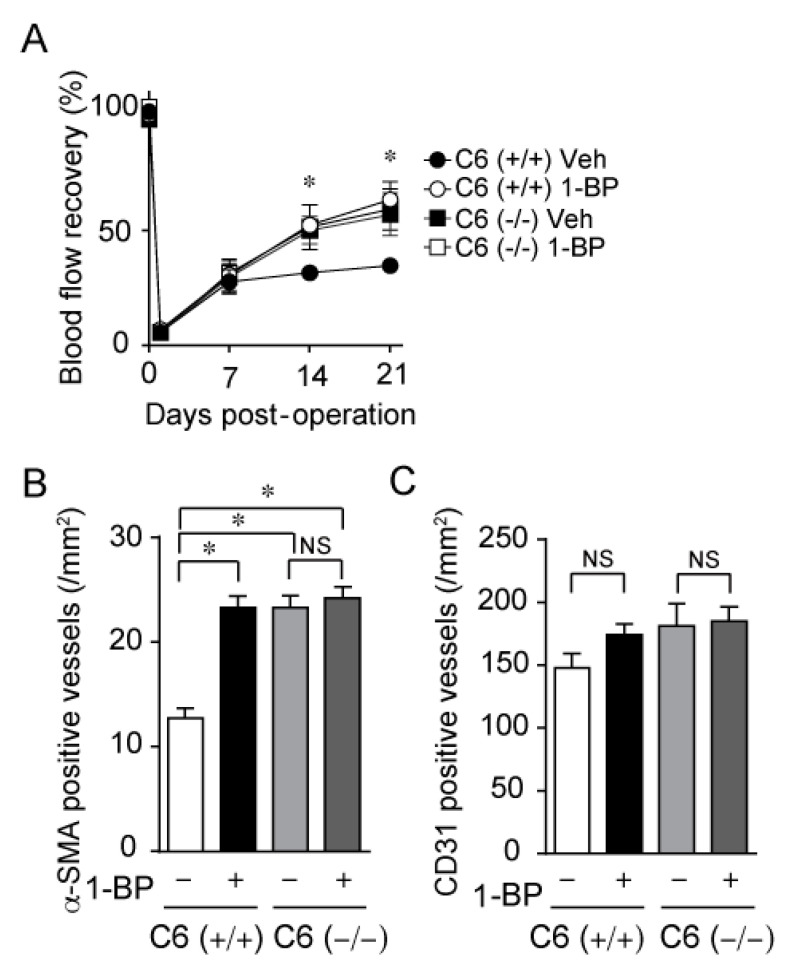
1-BP promotes arteriogenesis via TRPC6 inhibition. (**A**) Averaged time courses of blood flow recovery after HLI in 129/Sv wild type (C6 (+/+)) and TRPC6-knockout (C6 (−/−)) mice with 1-BP or vehicle (Veh) (n = 6 mice per genotype and treatment). (**B**) Quantitative result of the number of α-SMA positive vessels in either C6 (+/+) or C6 (−/−) ischemic gastrocnemii with or without 1-BP (n = 5 mice per genotype and treatment). (**C**) Quantitative result of the number of CD31 positive capillary in either C6 (+/+) or C6 (−/−) ischemic gastrocnemii with or without 1-BP (n = 5 mice per genotype and treatment). Data are mean ± s.e.m. * *p* < 0.05. Comparisons were made using Two-way ANOVA followed by Tukey’s multiple comparison tests (**A**) or One-way ANOVA followed by Tukey’s multiple comparison tests (**B**,**C**).

HLI also causes the disruption of skeletal muscles. Next, we evaluated the effect of 1-BP on the integrity of skeletal muscles by analyzing muscle atrophy and the abundance of myoglobin in gastrocnemius. Although HLI severely atrophy gastrocnemius muscle in both vehicle and 1-BP-treated groups, 1-BP-treated mice had larger CSA of individual skeletal muscle fibers than those that were vehicle-treated (Figure 6A). The abundance of myoglobin was significantly reduced 4 weeks after HLI in gastrocnemius of vehicle-treated mice. However, those were recovered in gastrocnemius of the 1-BP-treated group (Figure 6B and Figure S1). Consistent with these effects on morphology and protein quality in skeletal muscle tissue, motor activity represented by walking distance and velocity was improved by the administration of 1-BP (Figure 6C,D). These effects of 1-BP treatment on skeletal muscle recapitulated the effect of the gene knockout of TRPC6 (Figure 6E,F and Figure S1). We searched the factors which improve peripheral circulation after HLI and found that the expression of sphingosine kinase 1 (Sphk-1) was increased 2 weeks after HLI in gastrocnemius of 1-BP-treated mice (Figure 6G). The expression of Sphk-1 in gastrocnemius was also significantly increased 2 weeks after HLI in TRPC6-deficient mice (Figure 6H). At 2 weeks after HLI, either TRPC6-deficient or 1-BP-treated mice showed significant improvement of blood flow recovery (Figure 6A and Figure 7A). Direct comparison by parallel experiment demonstrated that the treatment of Pyr2 did not induce the recovery of both protein abundance of myoglobin and motor activity after HLI in contrast to that of 1-BP (Figure 6I–K and Figure S1). These data suggest that 1-BP facilitates the recovery of not only peripheral circulation but skeletal muscle mass.

### 3.5. 1-BP Improves Blood Flow via TRPC6 Inhibition

To investigate the underlying mechanism for the improvement of blood flow by 1-BP, we treated C6 (+/+) and C6 (−/−) mice with 1-BP. Our recent work demonstrated that genetic ablation of TRPC6 facilitates blood flow recovery after HLI (unpublished data). C6 (−/−) mice showed prominent blood flow recovery compared with C6 (+/+) mice (Figure 7A). In C6 (−/−) mice, treatment with 1-BP had no additional effect on peripheral blood flow recovery after HLI compared to vehicle treatment (Figure 7A). In contrast, 1-BP treatment significantly promoted blood flow recovery in C6 (+/+) mice to the level comparable to C6 (−/−) mice (Figure 7A). In addition, the recovery rates of peripheral blood flow were well-correlated with the degree of the number of α-SMA positive matured vessels (Figure 7B), but not with the number of CD31-positive vessels (Figure 7C). These data indicate that 1-BP improves blood flow via TRPC6 inhibition.

### 3.6. 1-BP Promotes Arteriogenesis Independently of Hypoxia and Inflammation

**Figure 8:**
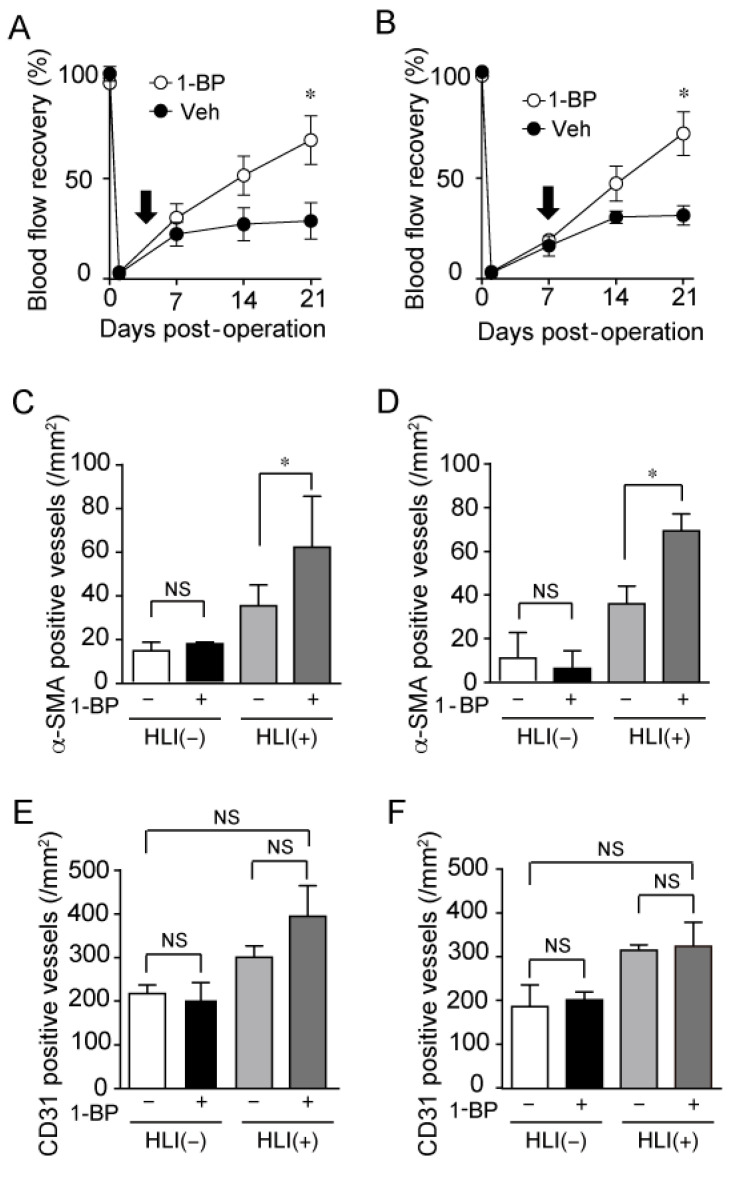
Treatment with 1-BP after HLI also promotes arteriogenesis and blood flow recovery. (**A**,**B**) Averaged time courses of blood flow recovery in mice treated with 1-BP or vehicle (Veh) after HLI. Osmotic pump including 1-BP (0.1 mg/kg/day) was implanted at 3 days (**A**) and 7 days after HLI (**B**), as shown by arrows (n = 8 mice). (**C**–**F**) Quantitative results of the number of α-SMA positive vessels (**C**,**D**) and CD31 positive vessels (**E**,**F**) at 3 weeks after HLI. (n = 3 mice). (**C**,**E**) 1-BP was administered from 3 days after HLI. (**D**,**F**) 1-BP was administered from 7 days after HLI. Data are mean ± s.e.m. (**A**,**B**) or median with 95% CI (**C**–**F**). * *p* < 0.05, NS.; no significance. Comparisons were made using Two-way ANOVA (**A**,**B**) or Kruskal-Wallis test (**C**–**F**).

In the early phase of blood flow recovery around 1 week after HLI, inflammation and hypoxic responses have critical roles in blood flow recovery [17]. To investigate the effect of 1-BP on inflammation and hypoxic factors, we administered 1-BP to HLI mice by implanting osmotic pump at day 3 and day 7 after HLI. Post-operative 1-BP treatment also significantly improved blood flow recovery (Figure 8A,B). The number of α-SMA positive vessels was significantly increased with 1-BP treatment (Figure 8C,D). Whereas CD31 positive capillaries had no remarkable change with 1-BP treatment (Figure 8E,F). These data indicate that 1-BP improves blood flow independently of initial events, such as hypoxia and inflammation, evoked right after arterial occlusion.

### 3.7. 1-BP Promotes Arteriogenesis Independently of Endothelium and Preserves Endothelial Function

**Figure 9:**
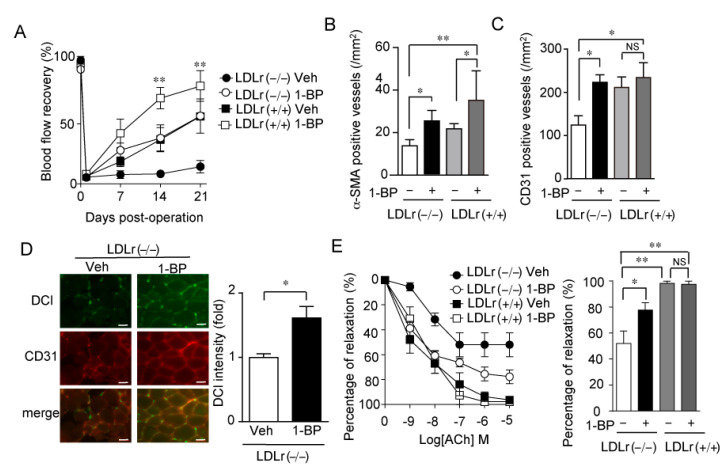
1-BP improves peripheral blood circulation in mice with endothelial dysfunction. (**A**) Averaged time courses of blood flow recovery in wild type (LDLr (+/+)) and LDLr-deficient (LDLr (−/−)) mice. Osmotic pump including 1-BP (0.1 mg/kg/day) or vehicle (Veh) was implanted 1 day before HLI (n = 4 mice per treatment and genotype). (**B**,**C**) Quantitative results of the number of α-SMA positive vessels (**B**) and CD31 positive vessels (**C**) at 21 days after HLI. (n = 4 mice). (**D**) Endothelium-dependent NO production detected by DCl-Da cal AM (10 nM) in CD31-positive vessels of ischemic gastrocnemii with 1-BP or Veh. Representative images (left) and quantitative result (right). Scale bars: 50 μm. (**E**) Effect of 1-BP on the endothelium-dependent relaxation of thoracic aorta. Aorta was first submaximally contracted by phenylephrine (10 μM) and then endothelium-dependent relaxation was induced by the indicated concentration of acetylcholine (ACh). Right, peak relaxation of aorta induced by 10^−5^ M ACh. Data are mean ± s.e.m. (**A**,**D**,**E**) or median with 95% CI (**B**,**C**). * *p* < 0.05, ** *p* < 0.01. Comparisons were made using Two-way ANOVA followed by Tukey’s multiple comparison tests (**A**), Kruskal-Wallis test (**B**,**C**), One-way ANOVA followed by Tukey’s multiple comparison tests (**D**) or Student t test (**E**).

We have previously reported that TRPC6 channel activity in VSMCs is suppressed by PKA- and PKG-dependent phosphorylation of TRPC6 at 69th threonine residue (Thr69) [14]. The NO delivery from vascular endothelium is an important endogenous mechanism for PKG-dependent phosphorylation of TRPC6 at Thr69. To test the involvement of endothelium function in the effect of 1-BP, we used LDLr (−/−) mice as an endothelial dysfunction model. LDLr (−/−) mice and wild type mice (LDLr (+/+)) were fed with a high fat diet for 1 month followed by HLI. LDLr (−/−) mice showed significantly reduced blood flow recovery, whereas this reduction was recovered by 1-BP treatment (Figure 9A). Matured vessel numbers in ischemic limbs were significantly increased with 1-BP in either LDLr (−/−) mice or LDLr (+/+) mice (Figure 9B). Vehicle-treated LDLr (−/−) mice showed a reduced number of CD31 positive capillaries (Figure 9C), NO production (Figure 9D), and endothelium dependent aortic relaxation (Figure 9E). Strikingly, 1-BP treatment recovered all of them (Figure 9C–E). These data indicate that 1-BP not only promotes the coverage of capillaries by VSMCs but also improves the endothelial function even in the endothelium dysfunction model.

## 4. Discussion

In this study, we identified the role of TRPC6 in regulation of blood flow recovery after HLI and the pharmacological effect of 1-BP as an inhibitor of TRPC3/6 for the blood flow recovery. Our results demonstrate that 1-BP treatment markedly improved blood flow after HLI by increasing the α-SMA positive blood vessels. Recently, we also reported that TRPC6 knockout mice showed better blood flow recovery after HLI than wild type mice. This facilitation of blood flow recovery in TRPC6 deficient mice were attributable to the increase in α-SMA positive vessels, consistent with the effect of 1-BP. We also demonstrated that there was no additive effect of 1-BP on the improved blood flow recovery of TRPC6-deficient mice, indicating that the pharmacological effect of 1-BP was brought by suppressing TRPC6. We have previously reported that suppression of TRPC6 in VSMCs facilitates differentiation [16]. As shown in Figure 6A, TRPC6 knockout mice have better blood flow recovery after HLI than WT mice. In our recent data, the phenotype of TRPC6 deficient mice could be reversed by VSMC-specific TRPC6 expression. Therefore, the target of 1-BP would be TRPC6 in VSMCs. Consistent with this, endothelial cell-mediated angiogenesis represented by the increase in CD31 immunofluorescence was not affected by 1-BP treatment. Strikingly, 1-BP treatment improved blood flow recovery even after surgery for HLI suggesting that 1-BP may be the promising agent for the treatment of PAD patients. In addition, 1-BP promotes arteriogenesis and blood flow recovery even under the condition of severe endothelial dysfunction.

Although several inhibitors of TRPC channels have been identified, there were no clinically-applicable drugs, yet probably because of the instability of the inhibitors in vivo or their possible toxic side effects. We have tested the effects of newly identified TRPC3/6 inhibitors using the HLI model. Among 7 inhibitors showing similar inhibitory action on TRPC3/6, 1-BP and Pyr2 were both effective to facilitate VSMC differentiation in vitro and also improve blood flow recovery after HLI in vivo. The extent of blood flow recovery by 1-BP was almost the same as that by broad TRPC channel inhibitor Pyr2. Although Pyr2 was known to suppress SOCE, an important process for immune responses [20,26], 1-BP has almost no effect on SOCE. We still do not know why other TRPC3/6 inhibitors had no beneficial effects on the PAD model mice. Taking the in vitro data of VSMC differentiation into consideration, it is unlikely that the difference among the TRPC3/6 inhibitory compounds is due to their in vivo pharmacokinetic properties. Therefore, 1-BP may have another effect on other cellular molecules, excluding TRPC6. Alternatively, this agent may allosterically modify the TRPC6 structure, which then affects downstream signaling in addition to the blocking of the TRPC6 gating mechanism. 

1-BP has beneficial effects on not only peripheral circulation but also skeletal muscle function. Myoglobin is released due to the ischemic injury of skeletal muscle. Even 4 weeks after HLI, myoglobin abundance remained lower in the ischemic leg than the control. 1-BP treatment preserves myoglobin abundance to comparable level of those in control. At the time point of 4 weeks after HLI, the blood flow recovery reaches a plateau, suggesting the tissue ischemia is already almost dissolved. Therefore, reduced myoglobin abundance in the ischemic leg implies that another important mechanism underlies skeletal muscle regeneration and recovery. Interestingly, Pyr2 exhibited a similar time course of blood flow recovery after HLI to that of 1-BP, but did not recover skeletal muscle quality and function as did 1-BP. These data suggest that 1-BP has a beneficial effect not only on vascular tissue but also skeletal muscle tissue during the recovery from ischemic tissue injuries. We have demonstrated that Sphk-1 expression in gastrocnemius was increased by HLI, and that both 1-BP treatment and TRPC6 deficiency further increased its expression compared to controls. Sphingosine 1-phosphate (S1P), a product of Sphk-1, reportedly promotes angiogenesis and arteriogenesis in the HLI model [27,28]. S1P has also been known to exert a trophic effect on skeletal muscle [29]. Therefore, the improvement of skeletal muscle quality and function by 1-BP would be mediated by the increase in Sphk-1 expression and production of S1P in recovering skeletal muscle tissue from ischemic injury.

Previous studies demonstrated the pivotal role of endothelial function on blood flow recovery in ischemia [30,31,32]. Hypercholesterolemia is one of the critical risk factors for PAD [33,34] and associates with poor angiogenesis and eventually lower peripheral perfusion in response to HLI [35,36,37]. The suppression of angiogenesis in response to tissue ischemia is reportedly attributed to the production of excess reactive oxygen species and reduced bioavailability of NO [38]. In this study, we demonstrated that 1-BP administration could improve blood flow recovery even in hypercholesterolemic mice. We also recently found that TRPC6-deficiency not only improves blood flow recovery but also increases the expression of endothelial NOS synthases in endothelial cells during HLI (unpublished data). Consistent with this, 1-BP increased the NO production during HLI. Therefore, 1-BP again recapitulate the effectiveness of genetic suppression of TRPC6.

A previous study has shown that Gα_13_ serves as a switch regulator of myofiber reprogramming through Rho-associated kinase 2-mediated pathway [39]. In another study, loss of USP21 increases myofiber type switch and thermogenesis [40]. In our additional qRT-PCR assays, we found decreases in *gna12* and *gna13,* and *usp21* mRNA levels in ischemic leg muscle (data not shown), which may have been due to general suppression of their constitutive expression levels. Hence, it seems that application of the hindlimb ischemic model may not be appropriate for the study of these targets and that the mechanistic basis of 1-BP may not be associated with myofiber type switch.

In summary, pharmacological suppression of TRPC6 by 1-BP has a huge potential as a new therapeutic strategy for PAD, a complicated vascular disease especially related to modern lifestyles.

## Data Availability

Not applicable.

## Supplementary Materials

The following supporting information can be downloaded at: https://www.mdpi.com/article/10.3390/cells11132041/s1, Figure S1: Uncropped western blot images.
