# Supplementary figures and images for: A TRPC3/6 Channel Inhibitor Promotes Arteriogenesis after Hind-Limb Ischemia
Source: Cells. 2022 Jun 27;11(13):2041. doi: 10.3390/cells11132041 (PMC9266111; doi:10.3390/cells11132041)

Figure 6B

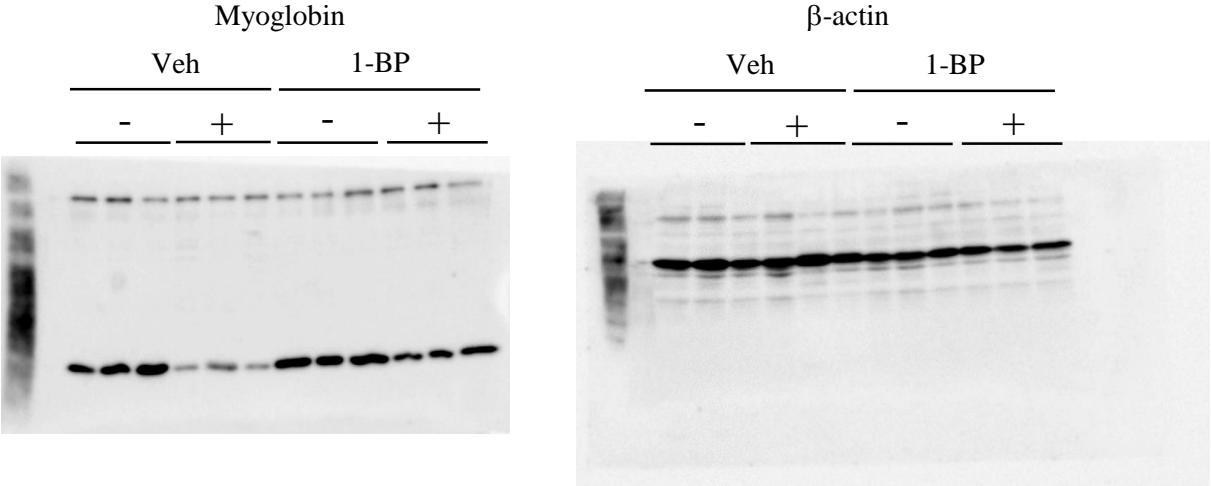

Figure 6F

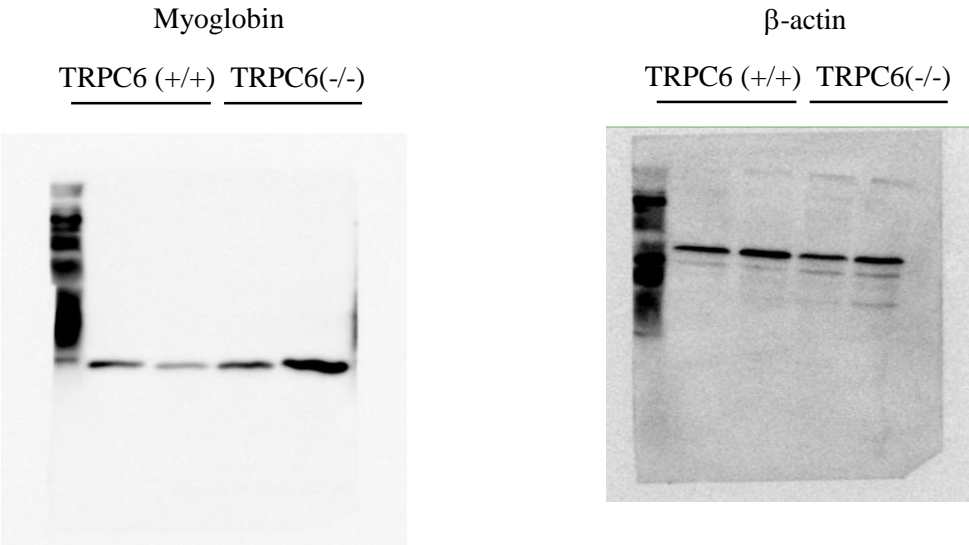

Figure 6I

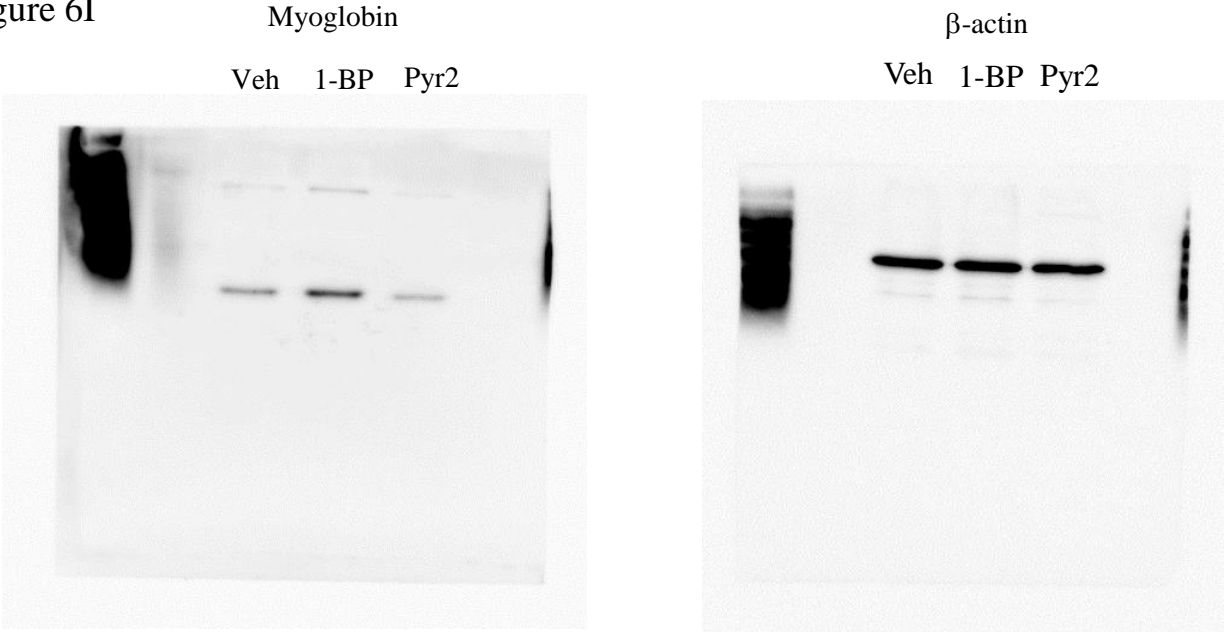

Supplement: Supplementary file 1 [file cells-11-02041-s001.zip › cells-1755128-Figure S1.pdf]
